# Effects of sexual assault vs. other traumatic experiences on emotional and cannabis use outcomes in regular cannabis users with trauma histories: moderation by gender?

**DOI:** 10.3389/fpsyg.2024.1386264

**Published:** 2024-05-31

**Authors:** Sherry H. Stewart, Juliana M. B. Khoury, Margo C. Watt, Pamela Collins, Sarah DeGrace, Pablo Romero-Sanchiz

**Affiliations:** ^1^Mood, Anxiety, and Addiction Comorbidity Lab, Department of Psychiatry, Dalhousie University, Halifax, NS, Canada; ^2^Department of Psychology and Neuroscience, Dalhousie University, Halifax, NS, Canada; ^3^Department of Psychology, University of Regina, Regina, SK, Canada; ^4^Department of Psychology, Saint Francis Xavier University, Antigonish, NS, Canada; ^5^School of Psychology, University of Sussex, Brighton, United Kingdom

**Keywords:** sexual assault, gender, PTSD, depression, hopelessness, cannabis use motives, cannabis craving, women

## Abstract

**Introduction:**

While sexual assault may have particularly adverse emotional effects compared with other forms of trauma, it remains unclear which emotional outcome dimensions are impacted, whether cannabis outcomes are similarly impacted, and whether gender differences exist in sexual assault’s links with these outcomes.

**Methods:**

*N* = 100 cannabis users with trauma histories (*M* age = 33.1) completed standardized measures of demographics, trauma exposure, posttraumatic stress (PTS) and depressive symptoms, hopelessness, and cannabis outcomes (frequency, medicinal prescription, motives, and craving).

**Results:**

Sexual assault was experienced more often by women (83.9%) than men (31.8%). A series of 2 × 2 analyses of variance [gender: women (*n* = 56) vs. men (*n* = 44) × trauma type: sexual assault (*n* = 61) vs. other (*n* = 39)] and logistic regression revealed that sexual assault survivors scored higher than other trauma survivors on re-experiencing and hyperarousal PTS symptoms (DSM-5 Clusters B and E), cognitive depressive symptoms, hopelessness, cannabis use frequency, medicinal cannabis prescription, cannabis use to cope with psychological symptoms, and compulsivity craving; and lower on social and enhancement cannabis use motives. In terms of gender main effects, women scored higher than men on cannabis use to cope with negative emotions. In terms of interactions for PTS Cluster D symptoms (negative alterations in mood/cognitions), among men only, sexual assault survivors scored higher than other trauma survivors; and for cannabis enhancement motives and purposefulness cannabis craving, among sexual assault survivors only, women scored higher than men.

**Discussion:**

Across many different trauma, women survivors’ use of cannabis to cope with negative affect should be a specific therapeutic focus. Moreover, we identified specific emotional and cannabis use outcomes that should be of specific clinical concern among sexual assault survivors regardless of gender. Finally, in terms of gender differences of clinical interest among sexual assault survivors, while PTS Cluster D symptoms should be specific treatment targets in men, cannabis enhancement motives and purposefulness craving should be treatment targets in women.

## 1 Introduction

Sexual assault is a form of trauma that is concerningly common around the globe (Dworkin et al., 2021). While definitions vary widely (Dworkin et al., 2021), sexual assault is generally considered to encompass any sexual acts (e.g., groping; kissing; anal, oral, or vaginal penetration) where at least one person did not consent or were coerced into participating through force or threat of harm (Breiding et al., 2015); this definition thus includes both childhood sexual abuse and sexual assault in adulthood. According to the U.S. Centers for Disease Control’s 2016/17 *Report on Sexual Violence*, 26.8% of women and 3.8% of men have been raped in their lifetimes (Basile et al., 2022). More broadly, 54.3% of women and 30.7% of men have experienced “contact sexual violence” (i.e., rape, being made to penetrate, sexual coercion, and/or unwanted sexual contact) in their lifetimes (Basile et al., 2022).

Not only is sexual assault common, but it also has numerous adverse emotional and behavioral sequelae in survivors. Indeed, sexual assault is explicitly included in the *Diagnostic and Statistical Manual of Mental Disorders, 5th edition* (DSM-5; American Psychiatric Association, 2013) definition of trauma [i.e., Criterion A for posttraumatic stress disorder (PTSD)]. Several meta-analyses have established links of moderate magnitude between sexual assault/childhood sexual abuse and PTS symptoms/PTSD diagnoses (e.g., Paolucci and Genuis, 2001; Dworkin et al., 2017; Dworkin, 2020). One meta-analysis suggests sexual assault has stronger links with PTS symptoms than with depression and anxiety symptoms (Dworkin et al., 2017), while other meta-analyses suggest similar magnitude links of sexual assault with PTS symptoms/PTSD diagnoses and depression (Paolucci and Genuis, 2001; Dworkin, 2020).

Theory suggests that unique aspects of sexual assault make it a particularly risky form of traumatic experience in terms of its potential to engender adverse emotional and behavioral outcomes (Dworkin et al., 2017). For example, sexual assault is a highly stigmatized type of trauma that is associated with significant myths (e.g., the victim is to blame for the assault); survivors often internalize this societal stigma, leading to shame, self-blame, and failure to seek help or disclose their experiences (Strickland et al., 2023). Substantial research supports that sexual assault is more strongly associated with PTS symptoms and PTSD diagnoses (e.g., Kessler et al., 1995; Kelley et al., 2009; Birkeland et al., 2021), and potentially also mood and anxiety disorders (Zinzow et al., 2008), than other forms of traumatic experience. The meta-analysis by Dworkin (2020) revealed that sexual assault was associated with a higher risk for lifetime and past-year PTSD, but not lifetime and past-year depression, than other types of trauma exposure. She suggested that more research is needed to determine more definitively whether sexual assault is more risky than other forms of trauma in terms of mental health consequences outside of PTSD since few studies addressing these other non-PTSD sequelae were available for meta-analysis.

Specific trauma characteristics may influence not only the overall levels of PTS symptoms but also the specific manifestation of PTS symptoms (Kelley et al., 2009; Grimm et al., 2012; Wilkinson et al., 2017). A study by Birkeland et al. (2021) examined PTS symptoms after sexual trauma compared to a variety of other traumatic events (i.e., accidents, family violence, bullying and/or threats, and sudden loss or serious illness of a loved one) in a large sample of treatment-seeking children and adolescents who identified their worst trauma. Not only was sexual trauma associated with more severe PTS symptoms overall than all other categories of the worst trauma examined, but it (along with bullying/threats and family violence exposure) was associated with relatively higher levels of negative beliefs and emotions. Birkeland et al. (2021) suggested that more work is needed to examine the specific pattern of posttraumatic sequelae observed following specific traumatic events (e.g., sexual assault); indeed, such work could help better target our treatment interventions to predominant presenting symptoms.

Given that sexual assault is associated with elevations in emotional disorder symptoms (e.g., PTS, depressive, and anxiety symptoms), it is perhaps not surprising that research suggests links between sexual assault exposure to substance use and substance use disorders. Indeed, substances are often misused as a maladaptive coping strategy for dealing with the emotional sequelae of traumatic experiences like sexual assault (Stewart, 1996; Stewart et al., 1998). Dworkin et al.’s (2017) meta-analysis showed a small but significant effect of sexual assault exposure on adverse substance use outcomes (*g* = 0.37). Similarly, in the meta-analysis of sexual assault effects on the odds of various psychiatric diagnoses, Dworkin (2020) found significantly increased odds of past-year substance use disorder among those with vs. without sexual assault histories (*OR* = 1.75). However, some research suggests that substance use disorder risk is not higher in those with sexual assault histories when compared to those who have experienced other traumas (Zinzow et al., 2008; Dworkin, 2020). Although most studies examine substances as a group, or separate alcohol from other drugs of abuse, some studies have examined the links of trauma exposure generally, and sexual assault specifically, to indices of cannabis use and cannabis use disorder (CUD). For example, trauma exposure, in general, has been associated with increased odds of cannabis use (Kevorkian et al., 2015) and regular cannabis use (Bassir Nia et al., 2023). Concerning studies that have specifically focused on sexual assault/childhood sexual abuse and cannabis outcomes, Abajobir et al. (2017) examined substantiated cases of childhood maltreatment and links to CUD 21 years later. They found that physical abuse, emotional abuse and neglect, as well as multiple episodes of maltreatment, but not sexual abuse, independently predicted CUDs in adulthood.

However, many experiences of childhood sexual abuse go unsubstantiated, making it important to include studies using self-report. Hayatbakhsh et al. (2009) found that self-reported childhood sexual abuse and rape before age 16 were each associated with increased odds of frequent cannabis use in emerging adulthood. Meyers et al. (2018) showed that a self-reported history of childhood sexual abuse was associated with an increased risk for CUD, even after controlling for a history of physical abuse and witnessing parental violence. Similarly, a recent meta-analysis by de la Peña-Arteaga et al. (2021) showed that childhood sexual abuse was associated with an increased risk for cannabis use in adolescence. Additional research specific to cannabis outcomes is needed since cannabis is frequently recommended as a strategy for managing the emotional sequelae of traumatic experiences like sexual assault (Talshir and Lavie-Ajayi, 2023) despite the lack of high-quality evidence for its efficacy in this regard (McKee et al., 2021). More work is also needed on the specific patterns and characteristics of cannabis use (e.g., frequency, cannabis prescription, motives, and patterns of craving) among those with sexual assault histories to test operant conditioning theory predictions regarding use for negative reinforcement.

Important gender differences exist in the rates and consequences of sexual assault. While women are less likely than men to experience many types of traumatic experiences (i.e., accidents, physical assault, disaster, combat/war, and witnessing death or injury), they are more likely to be sexually abused in childhood and sexually assaulted in adulthood (Tolin and Foa, 2006). Even though they are less likely than men to be exposed to traumatic events generally, women are more likely to meet the criteria for PTSD (Tolin and Foa, 2006). Women’s increased risk for PTSD may be at least partially due to their greater exposure to sexual assault, as this form of trauma carries one of the highest risks for PTSD of all traumatic events (Tolin and Foa, 2006). While women are more likely than men to experience PTSD following exposure to most types of traumatic experiences, this does not appear to be the case with sexual assault/childhood sexual abuse, where women and men are equally likely to develop PTSD following exposure (Tolin and Foa, 2006).

Looking across various emotional and behavioral outcomes of sexual assault exposure, the literature is quite mixed as to whether there are moderating effects of gender and, if so, whether sexual assault has particularly adverse effects for women or men. For example, in a study of university students, alcohol-involved sexual assault had more adverse emotional consequences for men than women survivors, which the authors attributed to the greater stigmatization of men survivors of sexual violence (Kehayes et al., 2019). Conversely, Sonmez et al. (2021) showed that sexual assault/childhood sexual abuse was more strongly associated with the severity of anhedonia (loss of interest/pleasure) in girls/women than boys/men in a sample of adolescents and emerging adults. Similarly, Hayatbakhsh et al. (2009) found that while childhood sexual abuse was associated with increased odds of frequent cannabis use in emerging adulthood among both men (*OR* = 2.1) and women (*OR* = 3.9), the odds were almost doubled in the women relative to the men. Similarly, in a study by Hébert et al. (2019), childhood sexual abuse was associated with a significantly increased likelihood of past-year cannabis use in high school, but only for girls. A meta-analysis of the links between interpersonal violence and psychological distress by Weaver and Clum (1995) also showed stronger links in samples with a greater proportion of women. However, their review included, but was not limited to, sexual assault and childhood sexual abuse. Meta-analyses were more specific to sexual assault and childhood sexual abuse by Dworkin et al. (2017) and Paolucci and Genuis (2001) did not find gender moderation of the effects of sexual trauma on the indices of psychopathology examined. Similarly, Meyers et al. (2018) showed that the adverse effect of childhood sexual abuse on CUD did not vary significantly by gender. Recently, Dworkin (2020) has called for an additional investigation of gender differences in the links between sexual assault with various emotional and behavioral outcomes, as the literature remains unclear as to the presence and direction of any gender differences.

While sexual assault is clearly linked with adverse emotional (e.g., PTS and depression) and behavioral (e.g., substance misuse) sequelae, several gaps remain in this literature. For example, while it has been established that sexual assault exerts more negative impacts on PTS symptoms than other forms of trauma exposure, the specific domains of PTS symptoms (i.e., intrusions, avoidance, negative impacts on mood/cognition, arousal) that are particularly impacted by sexual assault vs. other forms of trauma remain unclear. Moreover, it remains to be determined whether the particularly adverse impacts of sexual assault (vs. other trauma) are specific to PTS symptoms or if they extend to other types of emotional (e.g., depression and hopelessness) and behavioral (e.g., substance misuse) symptoms. Beyond research on childhood sexual abuse (e.g., Hayatbakhsh et al., 2009; Meyers et al., 2018), little research has focused on the associations of sexual assault with various cannabis use outcomes. Finally, the evidence is quite mixed regarding the presence and direction of gender moderation effects in the links of sexual assault with adverse emotional and behavioral outcomes, with some studies suggesting greater adverse effects in women (e.g., Sonmez et al., 2021), others suggesting greater adverse effects in men (e.g., Kehayes et al., 2019), and still, others suggesting no gender moderation (e.g., Dworkin et al., 2017), highlighting the need for more research.

The present study is a secondary analysis of data drawn from two lab-based studies of regular cannabis users with DSM-5-defined histories of trauma (i.e., Romero-Sanchiz et al., 2022; DeGrace et al., 2023, 2024). The purpose of the secondary analysis was to bridge the above-identified gaps in the literature. Specifically, we compared cannabis users who had been sexually assaulted with those who had experienced trauma other than a sexual assault on several emotional and behavioral outcomes, including four clusters of PTS symptoms, three clusters of depression symptoms, hopelessness, cannabis use frequency, medicinal cannabis prescription, four cannabis use motives, and four dimensions of cannabis craving. Since the combined sample represented a sufficiently large sample to examine moderators of the trauma type effects (sexual assault vs. other), we also examined if any effects of trauma type were moderated by gender.

We put forward five hypotheses and one exploratory research question. With respect to the frequency of sexual assault exposure, we hypothesized that consistent with prior work (e.g., Basile et al., 2022), a higher proportion of women than men in our sample would report personal exposure to sexual assault in their lifetimes (H1). With respect to gender main effects on emotional and cannabis outcomes, we expected that women would score higher on the emotional outcomes (PTS symptom subscales, depressive symptom subscales, and hopelessness) (H2), whereas men would score higher on the cannabis outcomes (i.e., cannabis use frequency, medicinal cannabis prescription, motives for use subscales, and craving subscales) (H3). With respect to the main effects of trauma type, we expected that those reporting exposure to sexual assault in their lifetime would score higher on all emotional outcomes (H4) and certain cannabis outcomes reflecting negative reinforcement learning [i.e., cannabis frequency, medicinal cannabis prescription, coping with negative emotions motives, coping with psychological symptoms motives, and relief cannabis craving scales (compulsivity and emotionality)] (H5). The positive reinforcement motives (i.e., social and enhancement) and reward craving subscales (i.e., expectancy and purposefulness; Romero-Sanchiz et al., 2022) were included as sensitivity tests for our hypotheses regarding trauma type main effects on the negative reinforcement motives and relief cannabis craving dimensions in H5. Given the mixed findings in the literature regarding whether sexual assault’s effects on various adverse outcomes are moderated by gender and, if so, if it is more strongly associated with adverse outcomes in men or women, we included potential interactions of gender and trauma type as an exploratory research question (RQ1) and did not make specific directional hypotheses.

## 2 Materials and methods

### 2.1 Participants

Participants were drawn from two lab-based studies examining trauma cue-elicited craving in regular cannabis users with trauma histories (i.e., Romero-Sanchiz et al., 2022; DeGrace et al., 2023, 2024). There were *n* = 51 and *n* = 50 participants in each study, respectively. Inclusion criteria for eligibility in both studies included regular cannabis use (at least weekly use),^1^ a lifetime history of exposure to at least one PTSD criterion A traumatic experience according to the DSM-5 (American Psychiatric Association, 2013) definition, being between 19 and 65 years old, and willingness to attend a lab-based testing session. Exclusion criteria for the parent studies were as follows: self-reports of taking medications that might numb responses to trauma or substance cue exposure (e.g., methadone), given the focus of the parent studies on in-lab reactivity to such cues and/or self-reports of severe mental illness (psychotic disorder or bipolar disorder). An additional exclusion for the present study was non-binary gender identity since the current study was focused on gender differences, and there were too few participants identifying beyond the traditional gender binary in the original samples to allow for reliable comparisons. One potential participant from the combined dataset was excluded on this basis, leaving a final combined sample of *N* = 100. On average, participants were 33.1 years of age, and 56% were women. Trauma characteristics of the final sample are shown in Table 1.

**Table 1:** Traumatic experiences reported in the total sample (*N* = 100).

Trauma:	Sexual assault (*n* = 61)	Other trauma (*n* = 39)	Total sample (*N* = 100)
Natural disaster	*n* = 18 (29.5%)	*n* = 17(43.6%)	*n* = 35 (35.0%)
Fire/Explosion	*n* = 19 (31.1%)	*n* = 9 (23.1%)	*n* = 28 (28.0%)
Transportation accident	*n* = 33 (54.1%)	*n* = 26 (66.7%)	*n* = 59 (59.0%)
Serious accident	*n* = 17 (27.9%)	*n* = 18 (46.2%)	*n* = 35 (35.0%)
Toxic exposure	*n* = 13 (21.3%)	*n* = 6 (15.4%)	*n* = 19 (19.0%)
Physical assault	*n* = 39 (56.5%)	*n* = 25 (64.1%)	*n* = 64 (64.0%)
Assault with weapon	*n* = 21 (34.4%)	*n* = 13 (33.3%)	*n* = 34 (34.0%)
Other unwanted sexual experience	*n* = 47 (77.0%)	*n* = 2 (5.1%)	*n* = 49 (49.0%)
Combat/War zone	*n* = 7 (11.5%)	*n* = 16 (15.4%)	*n* = 13 (13.0%)
Captivity	*n* = 10 (16.4%)	*n* = 2 (5.1%)	*n* = 12 (12.0%)
Life-threatening injury/illness	*n* = 12 (19.7%)	*n* = 11 (28.2%)	*n* = 23 (23.0%)
Extreme human suffering	*n* = 18 (29.5%)	*n* = 6 (15.4%)	*n* = 24 (24.0%)
Sudden violent death	*n* = 11 (18.0%)	*n* = 5 (12.8%)	*n* = 16 (16.0%)
Sudden accidental death	*n* = 6 (9.8%)	*n* = 5 (12.8%)	*n* = 11 (11.0%)
Caused serious injury/harm/death	*n* = 6 (9.8%)	*n* = 4 (10.3%)	*n* = 10 (10.0%)

Values represent the number (and column percent) who endorsed each category of a traumatic event in their lifetime on the Life Events Checklist for DSM-5 (LEC-5; Weathers et al., 2013). Other unwanted sexual experience = unwanted or uncomfortable sexual experience other than sexual assault. Data only presented for “happened to me.”

### 2.2 Measures

#### 2.2.1 Demographics

Participants completed an author-compiled demographics measure, which assessed gender (identification as a man, woman, or other gender identity) and age (in years).

#### 2.2.2 Trauma exposure

Participants were screened for trauma exposure with the Life Events Checklist for DSM-5 (LEC-5; Weathers et al., 2013). The LEC-5 lists a series of 16 potentially traumatic experiences (e.g., natural disaster, physical assault, and combat) according to DSM-5 Criterion A of a PTSD diagnosis (American Psychiatric Association, 2013). Participants checked all items representing traumatic events to which they had been exposed in their lifetimes (each dichotomously scored). Each item provided options for: happened to me, witnessed it, learned about it happening to someone close, and/or happened as part of my job consistent with the DSM-5 definition of a Criterion A event. This measure was used to determine study eligibility: respondents were only eligible for participation in either parent study if they endorsed at least one eligible potentially traumatic event on the LEC-5. This measure was also used to classify each participant based on their responses to LEC-5 item 8—sexual assault exposure which was defined as “rape, attempted rape, made to perform any type of sexual act through force of threat or harm.” Only those who endorsed this as having happened to them were placed in the sexual assault group (dichotomously scored as 0 = no; 1 = yes). The LEC-5 was also used to create the “trauma load” variable—a count of all non-sexual assault traumatic experiences the respondent endorsed as having happened to them in their lifetime (possible range = 0–15).^2^

#### 2.2.3 Posttraumatic stress symptoms

We used the Posttraumatic Stress Disorder Checklist for DSM-5 (PCL-5; Blevins et al., 2015) to assess PTS symptom severity. It consists of 20 items divided across four subscales, each corresponding to a PTS symptom cluster in the DSM-5 (American Psychiatric Association, 2013): re-experiencing (five items; present sample α = 0.782), avoidance (two items; α = 0.687), negative alterations in mood and cognitions (seven items; α = 0.870), and hyperarousal (six items; α = 0.734). Each item is rated on a scale ranging from 0 (not at all) to 4 (extremely). The PCL-5 has been shown to possess good psychometric properties (e.g., structural validity, test–retest reliability, convergent and discriminant validity) in previous studies (Blevins et al., 2015; Bovin et al., 2016); all four PCL-5 subscales showed adequate to good internal consistency in the present sample.^3^

#### 2.2.4 Depressive symptoms

We used the 21-item Beck Depression Inventory–II (BDI-II; Beck et al., 1996) to assess depressive symptom severity. Items are completed in relation to symptoms experienced in the past 2 weeks. Each answer is scored on a scale from 0 to 3, with higher scores indicating more severe depressive symptoms. Factor analytic studies suggest either two (affective–cognitive and somatic; e.g., Dozois et al., 1998) or three factors (affective, cognitive, and somatic; e.g., Vanheule et al., 2008). Given our desire to consider the affective and cognitive consequences of sexual assault separately, we scored the BDI-II according to three subscales (see Vanheule et al., 2008; O'Shea et al., 2018): affective symptoms (five items; e.g., sadness, pessimism, and loss of pleasure; present sample α = 0.711), cognitive symptoms (seven items; e.g., self-criticism, feelings of guilt, and worthlessness; α = 0.809), and somatic symptoms (nine items; e.g., loss of energy, changes in sleep, and changes in appetite; α = 0.750), all with adequate to good internal consistency in our sample.

#### 2.2.5 Hopelessness

We used the Hopelessness subscale of the Substance Use Risk Profile Scale (SURPS; Woicik et al., 2009). This scale consists of seven items tapping either the presence or absence of hopelessness. Participants rate the degree to which each item pertains to them on a scale from 1 (strongly disagree) to 4 (strongly agree). Following reverse scoring of the inversely keyed items, a total hopelessness score was obtained, with higher scores reflecting greater levels of hopelessness. Internal consistency was good in our sample (α = 0.871).

#### 2.2.6 Cannabis use frequency

An item is drawn from the Cannabis Use Disorder Identification Test–Revised (CUDIT-R; Adamson et al., 2010) assessed cannabis use frequency in the past 6 months. Response options ranged from *never* (coded as 0) to *daily or almost daily* (coded as 4).^4^

#### 2.2.7 Cannabis prescription status

Participants were asked to indicate whether they currently had a prescription for medicinal cannabis (dichotomously coded: no = 0; yes = 1).

#### 2.2.8 Cannabis use motives

Motives for cannabis use were assessed with three relevant subscales from the Marijuana Motives Measure (MMM; Simons et al., 1998): social (five items; present sample α = 0.812); enhancement (five items; α = 0.830), and coping (four items; α = 0.851). Given recent recommendations that studies of coping motives for cannabis use should also consider use to manage symptoms of an emotional disorder (Atasoy et al., 2023), we also included two author-compiled items at the end of the MMM as follows: “to manage psychological symptoms” and “to treat a psychological condition.” This new two-item scale showed good internal consistency (α = 0.805) and was moderately correlated but non-redundant with the MMM coping motives scale (*r* = 0.515, *p* < 0.001). We labeled this new two-item scale as “coping with psychological symptoms” and the original MMM coping subscale as “coping with negative affect” to distinguish the two. For all motives items, participants rated their relative frequency of use for each reason on a scale from 1 (*almost never/never*) to 5 (*almost always/always*).^5^

#### 2.2.9 Cannabis craving

Craving for cannabis was assessed with the 12-item Marijuana Craving Questionnaire–Short Form (MCQ-SF; Heishman et al., 2009), which is an abbreviated form of the original MCQ (Heishman et al., 2001). This measure taps current craving for cannabis across four three-item subscales: compulsive craving (i.e., inability to control use; present sample α = 0.818), emotionality craving (i.e., cannabis use in anticipation of relief from withdrawal or negative mood; α = 0.851), expectancy craving (i.e., anticipation of positive outcomes from cannabis use; α = 0.672), and purposefulness craving (i.e., intention to use cannabis for its positive effects; α = 0.907). Each item is rated on a scale ranging from *strongly disagree* (scored as 1) to *strongly agree* (scored as 7). Research attests to the good psychometric properties of the MCQ-SF, including acceptable internal consistency of the subscales, convergent validity with the original long-form MCQ, and support for the theorized four-factor structure (Heishman et al., 2009); the internal consistency of the four subscales in the present sample ranged from acceptable to excellent (see text footnote 3).

### 2.3 Procedure

Participants were community recruited from across the Halifax Regional Municipality through advertisements posted in the community (e.g., grocery stores), in local mental health clinics, on various social media platforms, and distributed through Veterans’ associations. Advertisements called for regular cannabis-using adults who had experienced one or more traumatic events in their lifetime to take part in an in-person research study examining links between trauma and cannabis use. Respondents to the ads were screened over the phone for eligibility, and those who were eligible were scheduled for in-person testing. In both parent studies, participants were asked to remain abstinent from psychoactive drugs for 12 h and from coffee for 2 h prior to lab testing. On the test day, participants first provided informed consent and then completed the study measures. While the MCQ-SF was used as an outcome measure in the cue exposure paradigm in the parent studies (Romero-Sanchiz et al., 2022; DeGrace et al., 2024), we only used the baseline (pre-cue exposure) version in the present study. Indeed, all measures utilized in the present study were completed prior to cue exposure in the parent studies.

### 2.4 Statistical analyses

For H1 (gender differences in sexual assault exposure), we conducted a 2 × 2 (gender × trauma type) Pearson’s chi-square to test whether a greater proportion of women than men in our sample had been exposed to sexual assault. For H2–H5 and RQ1, we conducted a set of 2 × 2 (gender × trauma type) Analyses of Variance (ANOVAs) on the continuous emotional and cannabis outcomes and a logistic regression for our one dichotomous cannabis outcome (medicinal cannabis prescription). Effects of interest included the main effects and interactions of gender (men vs. women) and trauma type (sexual assault vs. other). For main effects (H2–H5), all *p* values <0.05 were considered significant. For RQ1, an *a priori* decision was made to further probe all interactions at *p* < 0.10, given our specific interest in possible gender moderation of sexual assault effects on our study outcomes. This decision was based on recommendations that alpha can be relaxed in the case of interactions of interest given the increased power needed to detect interactions as compared to main effects (e.g., Winer, 1971) and since the mixed results in the literature regarding gender moderation led to us test interactions in an exploratory manner. Tests of simple effects were used to probe interactions for exploratory RQ1, and simple effects were only reported if they were significant at traditional *p* levels (*p* < 0.05). Effect sizes are reported for all analyses using Cohen’s *d* for mean comparisons and Cohen’s omega (ω) for comparisons of dichotomous outcomes. Cohen’s *d* was interpreted as 0.2 = small effect, 0.5 = medium effect, and 0.8 = large effect (Cohen, 1988); ω was interpreted as 0.1 = small effect, 0.3 = medium effect, and 0.5 = large effect (Cohen, 1988).

Sensitivity analyses were used to test the robustness of the findings reported in the main analyses. First, we tested age and “trauma load” (i.e., the total number of categories of non-sexual assault trauma endorsed on the LEC) as potential covariates. Since trauma load was a count variable, it was first tested for over-dispersion and transformations were made, if necessary, prior to analyses (Maindonald and Braun, 2007). If either age or trauma load showed gender or trauma type main effects or interactions in preliminary 2 × 2 (gender × trauma type) ANOVAs, we planned to re-run the main analyses with age or trauma load as a covariate to determine which effects in the main analyses remained statistically significant following control of confounds. Second, given several tests were performed, in a second set of sensitivity analyses, the Benjamini–Hochberg method (Benjamini and Hochberg, 1995) was used to control the false discovery rate (FDR). The FDR threshold was set at 0.05 within each model, such that there was a 5% chance that any finding was a false positive. Throughout the article, each *p* value is reported in its unadjusted format unless the FDR threshold is exceeded, in which case both adjusted and unadjusted *p* values are reported. This helped us caution readers about which effects in the main analyses could potentially be false discoveries due to multiple testing.

## 3 Results

### 3.1 Sexual assault exposure by gender

Of the total sample, 61% reported having been sexually assaulted in their lifetimes. A 2 × 2 (gender × trauma type) Pearson’s chi-square revealed that a significantly greater proportion of women (47/56 = 83.9%) than men (14/44 = 31.8%) reported a lifetime history of sexual assault on the LEC-5 [*χ^2^* (1) = 28.125, *p* < 0.001; ω = 0.53—a large effect size].

### 3.2 Emotional outcomes

Each of our continuous emotional outcomes was subject to a 2 × 2 (gender × trauma type) ANOVA to examine the main and interactive effects of gender and sexual assault, respectively. No main effects of gender emerged for any emotional symptom outcomes (i.e., the four dimensions of PTS symptoms, three dimensions of depressive symptoms, or hopelessness levels). The main effects of trauma type emerged for PCL-5 cognitive re-experiencing, PCL-5 hyperarousal, BDI-II cognitive symptoms, and SURPS hopelessness. In all cases, those who had been sexually assaulted scored higher on these emotional symptoms compared to those who had experienced trauma types other than sexual assault (see Table 2 for means and SEs by trauma type). All significant trauma-type effects were moderate in magnitude (*d*’s = 0.45 [hopelessness] to 0.61 [PTS re-experiencing]; see Table 2).

**Table 2:** Mean (±*SE*) emotional outcomes by trauma type (sexual assault vs. other).

Outcome	Sexual assault (*n* = 61)	Other trauma (*n* = 39)	*F* value	*p* value	Cohen’s *d* value
	Mean (*SE*)	Mean (*SE*)			
PTS: Re-experiencing	10.04 (0.60)	7.21 (0.75)	8.614	0.004^**^	0.605
PTS: Avoidance	5.25 (0.34)	4.43 (0.42)	2.304	0.132	0.311
PTS: Negative mood/cognition	15.11 (1.01)	12.22 (1.26)	3.197	0.077	0.367
PTS: Hyper-arousal	12.52 (0.77)	9.66 (0.96)	5.380	0.022^*†^	0.476
Depression: affective	4.63 (0.37)	4.01 (0.46)	1.122	0.292	0.215
Depression: cognitive	7.15 (0.61)	4.88 (0.76)	5.377	0.023^*†^	0.477
Depression: somatic	9.89 (0.69)	8.23 (0.86)	2.298	0.133	0.309
Hopelessness	17.26 (0.60)	15.17 (0.75)	4.723	0.032^*†^	0.446

PTS = Posttraumatic stress symptom clusters on PCL-5 (Blevins et al., 2015); Depression affective, cognitive, and somatic = BDI-II (Beck et al., 1996) subscales (O'Shea et al., 2018); Hopelessness = SURPS (Woicik et al., 2009). All *F*’s evaluated at df = 1, 96. ^*^p < 0.05, ^**^p < 0.01. For Cohen’s *d*, 0.20 = small effect, 0.50 = medium effect, and 0.80 = large effect. ^†^FDR threshold > 5% exceeded (PTS hyperarousal: adjusted *p* = 0.066; Depression cognitive: adjusted *p* = 0.069; Hopelessness: adjusted *p* = 0.096) indicating potential false positive findings.

**Figure 1:**
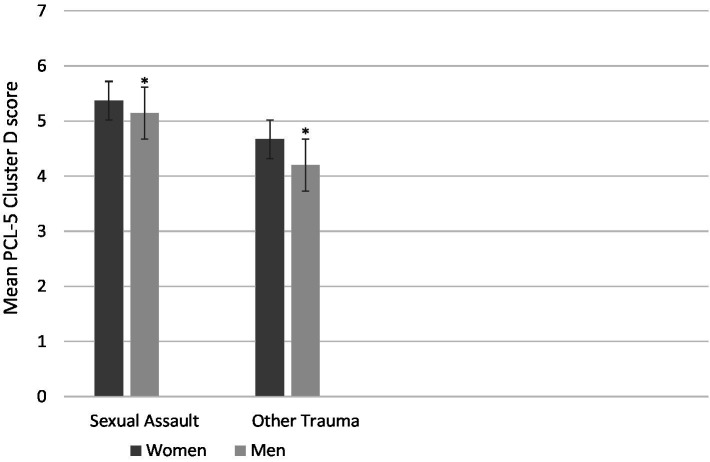
Mean posttraumatic stress cluster D symptom severity on the PCL-5 as a function of Trauma Type (sexual assault vs. other trauma) and Gender (women vs. men). There is a significant simple effect of Trauma Type in the men (*p* = 0.008; Indicated with asterisks) but not the women (*p* = 0.979). Bars represent standard errors.

With respect to the exploratory analyses of gender by trauma type interactions, a marginal interaction emerged (*p* = 0.071) for PCL-5 negative alterations in mood and cognitions. When probed using analyses of simple effects (see Winer, 1971), a simple main effect of trauma type was revealed for men (*p* = 0.008; *d* = 0.88—a large effect) but not for women (*p* = 0.979); men who had been sexually assaulted reported greater negative alterations in mood and cognition than men who had experienced trauma that did not include sexual assault (see Figure 1).

### 3.3 Cannabis outcomes

Each of our continuous cannabis outcomes (i.e., cannabis frequency, the four cannabis use motives, and the four dimensions of cannabis craving) was subject to a 2 × 2 (gender × trauma type) ANOVA to examine the main and interactive effects of gender and sexual assault, respectively. The only significant gender main effect on any of the cannabis variables was for women [*M* (*SE*) = 15.16 (0.83)] to score higher than men [*M* (*SE*) = 12.78 (0.73)] in the case of cannabis negative affect coping motives [*F* (1, 96) = 4.645, *p* = 0.034; *d* = 0.43—a medium effect size]. The main effects of trauma type emerged for cannabis use frequency, cannabis compulsivity craving, and cannabis use to cope with psychological symptoms. Those who had been sexually assaulted scored higher on these cannabis outcome measures compared to those who had experienced other types of trauma (see Table 3 for means and SEs by trauma type). Interestingly, for the two positive reinforcement motives for cannabis use on the MMM (i.e., social and enhancement motives), the main effects of trauma type also emerged. In these two cases, those who had been sexually assaulted scored *lower* than those who had experienced other types of trauma (see Table 3 for means and SEs by trauma type). All significant trauma-type effects were moderate in magnitude (*d*’s = 0.42 [compulsive craving] to 0.67 [symptom coping motives]; see Table 3).

**Table 3:** Mean (±*SE*) cannabis outcomes by trauma type (sexual assault vs. other).

Outcome:	Sexual assault (*n* = 61)	Other trauma (*n* = 39)	*F* value	*p* value	Cohen’s *d* value
	Mean (*SE*)	Mean (*SE*)			
Cannabis use frequency	3.91 (0.10)	3.57 (0.13)	4.541	0.036^*†^	0.494
Negative affect coping motives	14.36 (0.69)	13.59 (0.86)	0.484	0.488	0.143
Enhancement motives	14.60 (0.80)	18.08 (1.00)	7.389	0.008^**^	0.557
Social motives	10.56 (0.77)	13.58 (0.96)	6.039	0.016^*^	0.502
Symptom coping motives	7.62 (0.47)	5.49 (0.60)	7.772	0.007^**^	0.647
Compulsive craving	6.44 (0.56)	4.62 (0.70)	4.128	0.045^*†^	0.416
Emotionality craving	10.66 (0.74)	10.86 (0.92)	0.028	0.867	0.035
Expectancy craving	13.58 (0.69)	13.38 (0.86)	0.035	0.853	0.037
Purposeful craving	10.51 (0.80)	12.28 (1.00)	1.885	0.173	0.283

Cannabis use frequency = single item from the Cannabis Use Disorder Identification Test–Revised (CUDIT-R; Adamson et al., 2010); Negative affect coping, enhancement, and social motives = Marijuana Motives Measure (MMM) subscales (Simons et al., 1998); Craving Dimensions = subscales of the Marijuana Craving Scale–Short Form (MCQ-SF; Heishman et al., 2009); Cannabis Prescription = author-compiled single item dichotomous measure; Symptom Coping Motives = two items author-compiled measure added to the end of MMM; All *F*’s evaluated at df = 1, 96 save cannabis use frequency (*n* = 79) and symptom coping motives (*n* = 81). ^*^p < 0.05, ^**^p < 0.01; ^***^p < 0.001. For Cohen’s *d*, 0.20 = small effect, 0.50 = medium effect, and 0.80 = large effect. ^†^FDR threshold > 5% exceeded (Cannabis use frequency: adjusted *p* = 0.108; Compulsive craving: adjusted *p* = 0.135) indicating potential false positive findings.

We also conducted a logistic regression analysis on the dichotomous cannabis prescription variable using gender (men vs. women), trauma type (sexual assault vs. other), and their interaction as potential predictors, which were entered in a forward stepwise fashion. In the first step, trauma type was a significant predictor [*χ^2^* (1) = 9.299, *p* = 0.002], with those in the sexual assault group being significantly more likely to have a cannabis prescription (*n* = 36/61 = 59.0%) than those in the other trauma group (*n* = 11/39 = 28.2%) (ω = 0.31—a medium effect). An equation involving a sexual assault group predicting cannabis prescription status represented the final model [Wald (df = 1) = 8.679, *p* = 0.003] since neither the addition of gender nor the addition of the gender x trauma group interaction significantly improved prediction accuracy.

**Figure 2:**
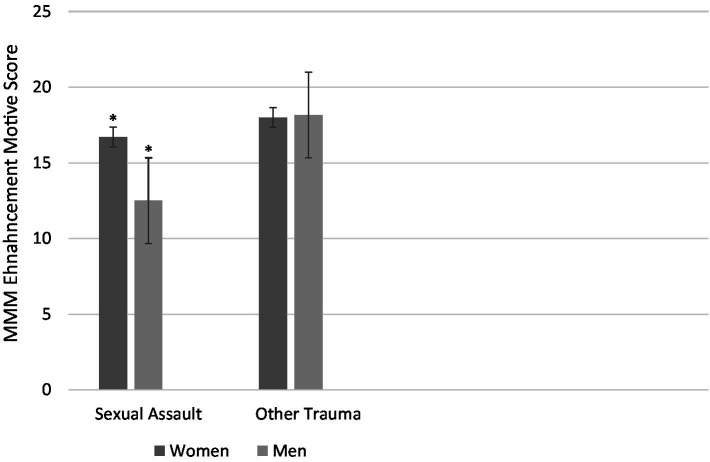
Mean enhancement motives for cannabis use on the MMM as a function of Trauma Type (sexual assault vs. other trauma) and Gender (women vs. men). There is a significant simple effect of Gender in the sexual assault group (*p* = 0.010; indicated with asterisks) but not the other trauma group (*p* = 0.934). Bars represent standard errors.

**Figure 3:**
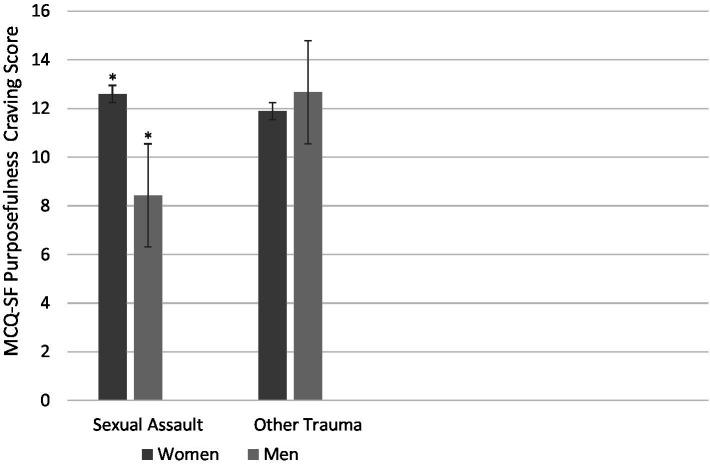
Mean purposefulness craving for cannabis on the MCQ-SF as a function of Trauma Type (sexual assault vs. other trauma) and Gender (women vs. men). There is a significant simple effect of Gender in the sexual assault group (*p* = 0.011; indicated with asterisks) but not the other trauma group (*p* = 0.699). Bars represent standard errors.

With respect to our exploratory analyses of potential interactions between gender and trauma type for the cannabis outcomes, marginal interactions emerged in the cases of two reward/positive reinforcement cannabis outcomes: cannabis purposefulness craving on the MCQ-SF and enhancement motives for cannabis use on the MMM (*p* = 0.058 and *p* = 0.091, respectively; see Figures 2, 3). When probed using analyses of simple effects (see Winer, 1971), a consistent pattern emerged on both measures. Specifically, a simple main effect of gender (higher in women than men) was revealed for those who had experienced sexual assault (*p* = 0.011 and *p* = 0.010 (*d* = 0.79 and *d* = 0.80—both large effects) for purposefulness craving and enhancement motives, respectively), but not for those who experienced trauma types other than sexual assault (*p* = 0.699 and *p* = 0.934 for purposefulness craving and enhancement motives, respectively).

### 3.4 Sensitivity analyses

We used a count variable of the number of non-sexual assault traumas personally experienced as an index of “trauma load.” The mean number of non-sexual assault traumas experienced in the total sample was 4.32 (variance = 7.27). This count variable displayed overdispersion (e.g., variance larger than the mean), indicating that it was not suitable for analysis in a factorial ANOVA. Thus, we first square root transformed the trauma load variable prior to subjecting it to a 2 × 2 (gender × trauma type) ANOVA (Maindonald and Braun, 2007). No significant main effects or interactions of gender or trauma type were observed, suggesting no need to covary this variable in sensitivity analyses.

We then ran a 2 × 2 (gender × trauma type) ANOVA on age to determine whether age should be controlled in a supplemental sensitivity analysis. There were gender and trauma type differences in age {men older than women [*F* (1, 96) = 6.986, *p* = 0.010]; men *M* (*SE*) age = 36.79 (1.84) vs. Women *M* (*SE*) age = 29.48 (2.07); sexual assault survivors older than other trauma victims [*F* (1, 96) = 5.965, *p* = 0.016]; sexual assault group’s *M* (*SE*) age = 36.52 (1.73) vs. other trauma group’s *M* (*SE*) age = 29.76 (2.16)}. We correlated age with all study outcome variables as a second step in determining in what cases age needed to be controlled as a covariate in our sensitivity analyses. The purpose was to determine if the effects identified in the main analyses reported above persisted when controlling for the potential confound of age. Age was significantly negatively correlated with MMM social (*r* = −0.317, *p* = 0.001) and enhancement motives for cannabis use (*r* = −0.307, *p* = 0.002). The 2 × 2 ANOVAs on the two motives variables from the primary analyses reported above were re-run using age as a covariate. The significance and direction of all effects (and relevant simple effects) for enhancement motives remained consistent with those reported above. However, the significant main effect of trauma type on social motives (sexual assault < other trauma) was eliminated when controlling the potentially confounding influences of age. Age was also significantly positively correlated with having a medicinal cannabis prescription (*r* = 0.290, *p* = 0.003). Thus, we ran a supplementary logistic regression analysis predicting the dichotomous cannabis prescription outcome with trauma type (sexual assault vs. other) as a predictor and age as a covariate. Together, the two variables significantly predicted the likelihood of having a medicinal cannabis prescription [*χ^2^* (2) = 16.76, *p* < 0.001], with both trauma type [Wald (df = 1) = 7.57, *p* = 0.006; sexual assault > other trauma] and greater age [Wald (df = 1) = 6.60, *p* = 0.010] independently predicting an increased likelihood of having a medicinal cannabis prescription; thus the trauma type effect on having a medicinal cannabis prescription persisted when controlling age.

The final sensitivity analysis made use of the FDR of Benjamini and Hochberg (1995) to identify effects that could potentially have been false discoveries due to multiplicity testing. While the significant main effects of trauma type on PTS re-experiencing, medicinal cannabis prescription, and coping with psychological symptoms motives, social motives, and enhancement motives for cannabis use survived this correction, the significant main effects of trauma type reported for PTS hyperarousal, cognitive depression symptoms, hopelessness, cannabis use frequency, and compulsive cannabis craving were identified as potential false discoveries (see Tables 2, 3). Similarly, the one significant main effect of gender observed in the main analyses for coping with negative affect motives for cannabis use was identified as a potential false discovery secondary to multiplicity testing (adjusted *p* = 0.102).

## 4 Discussion

The purposes of the present study were as follows: (a) to compare cannabis users who were sexual assault survivors to cannabis users with histories of other forms of trauma to determine whether sexual assault survivors have particularly harmful outcomes in terms of emotional and cannabis use variables and (b) to determine whether the links of sexual assault victimization (vs. other types of trauma exposure) with emotional and cannabis use outcomes are moderated by gender. For emotional outcomes, we examined the four domains of PTS symptoms (re-experiencing, avoidance, negative beliefs/emotions, and hyperarousal), the three domains of depressive symptoms (affective, cognitive, and somatic), and hopelessness. For cannabis use outcomes, we examined cannabis frequency, medicinal cannabis prescription, four motives dimensions, and four craving dimensions. Partially consistent with hypotheses, sexual assault survivors scored higher than other trauma survivors on re-experiencing and hyperarousal PTS symptoms, cognitive depressive symptoms, and hopelessness (emotional outcomes), and cannabis frequency, medicinal cannabis prescription, cannabis use to cope with psychological symptoms, and compulsivity craving (cannabis outcomes) (all moderate effects). Although not hypothesized, sexual assault survivors also scored *lower* on the positive reinforcement cannabis use motives (social and enhancement)—both moderate effects. For the PTS negative alterations in mood/cognitions cluster, sexual assault survivors scored higher than other trauma survivors only among men (a large effect). For cannabis enhancement motives and purposefulness craving, women scored higher than men only among sexual assault survivors (both large effects).

In terms of gender differences in sexual assault experiences, consistent with substantial prior research, the women in our sample were much more likely to report lifetime exposure to sexual assault on the LEC-5 (Weathers et al., 2013), replicating prior findings (Tolin and Foa, 2006) in regular cannabis using sample. Indeed, there was a 2.6-fold increase in the likelihood of sexual assault among women vs. men in our sample, and the effect was large in magnitude. However, sexual assault was still concerningly prevalent among the men, with a lifetime history reported by almost one-third of men in our sample, which is substantially elevated relative to the rate of 8% previously reported for men in the general Canadian population (Cotter and Savage, 2019).

We hypothesized that we would replicate prior findings that sexual assault is associated with more severe PTS symptoms than other forms of trauma (Dworkin, 2020) in a sample of regular cannabis users. Consistent with calls for additional research to identify which specific PTS symptoms are particularly associated with sexual assault (Birkeland et al., 2021), but to overcome the limitations of prior research that examined this issue at the level of unreliable single items (Kelley et al., 2009), we examined which of the four specific PTS symptom clusters on the PCL-5 differed by trauma group. We found that both re-experiencing and hyperarousal PTS symptom clusters (DSM-5; American Psychiatric Association, 2013; Criteria B and E, respectively) were significantly higher in sexual assault survivors than among survivors of other trauma. Given the role of fear conditioning in the intrusive symptom clusters of re-experiencing traumatic memories and hyperarousal (Careaga et al., 2016), these results suggest that fear conditioning may be particularly strong in sexual assault, potentially because it is often a sudden onset traumatic event during which survivors may perceive a threat to their lives (Wilkinson et al., 2017). However, only the re-experiencing and not the hyperarousal trauma type effect survived the FDR correction for multiplicity testing, cautioning that our trauma type effect on PTS hyperarousal symptoms may be a potential false discovery and thus requires replication.

We also wished to contribute to the literature on whether the adverse emotional consequences of sexual assault relative to other forms of trauma are specific to PTS symptoms by examining dimensions of depressive symptoms (affective, cognitive, and somatic) and a depression risk factor, namely hopelessness (Woicik et al., 2009). Prior research has been mixed as to whether sexual assault is associated with increased depressive symptoms when compared to other forms of trauma, with some studies supporting this specific link (e.g., Zinzow et al., 2008) and others contesting it (e.g., Dworkin, 2020). Our findings showed that cannabis users with sexual assault histories did suffer greater cognitive symptoms of depression (e.g., thoughts of past failure, worthlessness, and self-criticism) but not greater affective or somatic depressive symptoms on the BDI-II than cannabis users with histories of other trauma. Prior inconsistencies in the literature on whether sexual assault is associated with higher levels of depressive symptoms relative to other forms of trauma may have been due to a failure to consider the multidimensional nature of depression. Alternatively, this pattern of findings might be explained by the overlap in the cognitive symptoms of depression and PTS symptoms (Dworkin et al., 2017). We also showed that sexual assault survivors scored higher than those with other forms of traumatic experience in levels of hopelessness on the SURPS (i.e., negative beliefs about the self, the world, and the future). Together, these results for cognitive depressive symptoms and hopelessness suggest that sexual assault is a form of trauma that is particularly likely to engender negative beliefs about oneself, potentially due to the internalization of societal myths regarding sexual assault and associated self-blame (Strickland et al., 2023). However, neither the depressive cognitive symptoms nor the hopelessness trauma-type effects survived the FDR correction for multiplicity testing, cautioning these two effects may be potential false discoveries and thus require replication.

We had also hypothesized that, consistent with operant conditioning theory, sexual assault survivors would show higher cannabis use frequency, greater rates of medicinal cannabis prescription, higher negative reinforcement motives for cannabis use (coping with negative emotions and coping with psychological symptoms), and greater relief cannabis craving (compulsivity and emotionality craving; Romero-Sanchiz et al., 2022) than survivors of other forms of trauma exposure. Partially consistent with these predictions, sexual assault survivors did show higher cannabis use frequency, greater rates of medicinal cannabis prescription, greater cannabis use to cope with psychological symptoms (but not to cope with negative emotions generally), and greater compulsivity cannabis craving (but not greater emotionality craving). The greater susceptibility of sexual assault survivors to PTS re-experiencing and hyperarousal symptoms may create circumstances that present greater opportunities for negative reinforcement learning based on experiences of cannabis relieving these intrusive symptoms. This could lead to greater motivations to use cannabis to cope with these intrusive symptoms, seeking medical prescriptions for cannabis, and, consequently, greater frequency of use and difficulty controlling use (i.e., compulsivity craving). It is interesting that those with sexual assault histories did not report greater use of cannabis to manage negative affect on the MMM coping motives subscale, suggesting their elevated coping motives were quite specific to managing their more severe symptoms of emotional disorders. Also, while it is unexpected that sexual assault survivors did not report higher levels of emotionality craving (i.e., desire to use cannabis to manage aversive states), the items on this scale may have been too general to craving cannabis to manage negative emotions rather than to manage emotional disorder symptoms. Alternatively, given the craving measure used was a state craving tool and we used baseline craving values from the parent studies, it may be necessary to activate emotionality craving through experimental means such as a trauma cue reactivity paradigm (DeGrace et al., 2022) to observe sexual assault (vs. other trauma) effects on this cannabis outcome. It is important to note that only the symptom coping motives and cannabis prescription trauma type effects, and not the cannabis use frequency nor the compulsivity craving trauma type effects, survived the FDR correction for multiplicity testing, cautioning that our trauma type effects on the latter two cannabis outcomes may be potential false discoveries and thus require replication.

We included the positive reinforcement motives (social and enhancement) and the reward cannabis craving scales (purposefulness and expectancy) from the MMM and the MCQ-SF, respectively, as control measures to show the specificity of the hypothesized sexual assault group elevations to the negative reinforcement motives and relief craving dimensions of cannabis-related cognitions. However, some unexpected findings emerged for cannabis use motives. Specifically, those with sexual assault histories scored significantly *lower* on the two positive reinforcement motives (i.e., social and enhancement motives) than those who survived other forms of trauma exposure; the trauma type effects for both positive reinforcement motives survived stringent multiplicity correction, and the trauma type effect for enhancement motives persisted even when controlling age differences across trauma groups. Perhaps the avoidance behavior typical of PTSD in sexual assault survivors may involve avoidance of the types of contexts where enhancement-motivated cannabis use most often takes place (e.g., at bars; Shrier et al., 2013) if these serve as reminders of survivors’ traumatic experience. Regardless, it is very interesting that not only are sexual assault survivors more motivated than survivors of other forms of trauma to use cannabis to cope with psychological symptoms, but they also are less likely to use cannabis to enhance positive mood.

We were concerned that any differences between those with sexual assault histories and those with histories of other traumatic experiences only on our emotional or cannabis outcomes might be due to a greater history of non-sexual assault trauma among those in the sexual assault group compared to the other trauma control group. Indeed, prior research has shown that the risk of sexual assault is increased among those with prior trauma histories (Acierno et al., 1999). For this reason, we calculated a “trauma load” variable from the LEC-5, which represented the number of different categories of non-sexual assault trauma experienced. Gender by trauma type analyses of the trauma load variable provided no evidence that sexual assault survivors in our sample had a greater trauma load than other trauma group controls. Thus, it appears unlikely that the effects which we are attributing to sexual assault histories may be simply due to greater cumulative experiences of trauma in the sexual assault group.

Unexpectedly, we found very few main effects of gender in our sample of cannabis users in either emotional disorder symptoms or on our cannabis outcome variables. While substantial research suggests that women are at increased risk of PTSD from various traumatic experiences (Tolin and Foa, 2006) and that women are at increased risk of depression (Nolen-Hoeksema and Girgus, 1994), we saw no evidence of women scoring higher than men on any dimension of PTS symptoms or any of the other emotional outcome measures (depressive symptom domains, hopelessness). It is possible that these gender differences do not extend to cannabis users or that the gender effects were too small in magnitude to be detected in our sample of *N* = 100. Similarly, while substantial research suggests that men use cannabis more frequently than women (Crocker and Tibbo, 2018), we saw no evidence of greater scores among men on any of our cannabis outcomes. This pattern is consistent with convergence (i.e., women increasing their cannabis use levels to reach levels seen in men; Chapman et al., 2017); convergence is of particular concern in the post-legalization context in Canada, where recreational cannabis use has been legal since 2018 (Matheson and LeFoll, 2023). Indeed, the only main effect of gender in the current study was for cannabis coping motives on the MMM, with women cannabis users with trauma histories scoring *higher* than their men counterparts. This is consistent with recent results reported by Phillips (2021) that women cannabis users reported greater negative affect reduction motives for use than men cannabis users did. Given that cannabis users who report greater levels of coping with negative affect motives have been shown to be at higher risk of experiencing psychosocial harm associated with their use (e.g., Buckner, 2013; Foster et al., 2016), our findings suggest that women cannabis users with trauma histories may represent an at-risk group who may be particularly susceptible to adverse outcomes of their cannabis use. However, this gender main effect did not survive FDR correction for multiplicity testing, suggesting the possibility that it may be a false discovery and emphasizing the need for replication.

A final area of contribution of the current study pertains to whether the emotional and cannabis use effects of sexual assault compared to other forms of trauma are moderated by gender. We found three marginal interactions of trauma type with gender, which, when probed, revealed an interesting pattern of significant simple main effects. First, for the negative alterations in mood/cognition domain of PTS symptoms (i.e., DSM-5; American Psychiatric Association, 2013; Criterion D), sexual assault survivors scored higher than other trauma survivors, but only among men. This finding is partially consistent with the findings of Birkeland et al. (2021) in adolescents, showing that sexual trauma (along with bullying/threats and family violence exposure) was associated with relatively higher levels of negative beliefs and emotions and consistent with Kehayes et al.’s (2019) finding that the negative emotional consequences of alcohol-involved sexual assault in university students were particularly strong among men as compared to women survivors. This finding suggests that the development of maladaptive beliefs (e.g., self-blame) and emotions (e.g., guilt) following sexual assault vs. other forms of trauma may be particularly likely among men survivors, perhaps because of the enhanced societal stigma toward sexual assault in men survivors and/or the increased reluctance of men to disclose such experiences (Kehayes et al., 2019).

The other two gender moderation effects revealed that women scored higher than men, but only among sexual assault survivors, on two cannabis outcomes pertaining to putative positive reinforcement/reward processes. Specifically, women sexual assault survivors reported greater enhancement motives for cannabis use (reasons for use involving enhancement of pleasure) and more purposefulness of cannabis craving (i.e., intentional planning to use cannabis for its pleasurable outcomes). Previous work has shown that the link between sexual assault and anhedonia (absence of pleasure) is stronger in women than men (Sonmez et al., 2021). Perhaps their greater anhedonia motivates women sexual assault survivors’ greater intentional, planful use of cannabis as a means of self-medication for this loss of pleasure, given cannabis’ euphoric effects.

The present study’s findings should be interpreted with several potential limitations in mind. First, as a secondary data analysis, the present study was limited by the measures included in the parent studies. While the present study benefitted from the inclusion of common measures of several emotional and cannabis use outcomes in the two original studies, unfortunately, no common measures of anxiety or anhedonia were available. Second, there are limitations to the LEC-5 (Weathers et al., 2013) as a trauma measure: although we excluded item 9 (other unwanted/uncomfortable sexual experiences) from our conceptualization of sexual assault, given how common it is for survivors to minimize their experiences, less “serious” assaults (e.g., groping) may have been reported in this “other” category that was intended for sexual harassment experiences. Moreover, the LEC-5 taps lifetime sexual assault, meaning that childhood sexual abuse cannot be separated from adult sexual assault despite their differing characteristics (Wilkinson et al., 2017). Third, as this was a cross-sectional study, it is not possible to determine the causality or even directionality of the sexual assault effects. Indeed, research shows that sexual assault can act as both a risk factor for and an outcome of psychiatric symptoms like PTS and substance misuse (e.g., Xu et al., 2013). Fourth, the sample size available for analysis (*N* = 100) did not provide adequate power for detecting smaller gender × trauma type interactions, and the three interactions reported were only marginally significant in the omnibus ANOVAs, suggesting they require replication in a larger independent sample. Fifth, several statistical tests were performed, increasing the possibility of chance findings. Sensitivity analyses correcting for multiplicity testing using the FDR of Benjamini and Hochberg (1995) revealed that some of the significant effects reported in the main analyses could be false discoveries and thus require independent replication with increased statistical power. Sixth, one of our measures (cannabis use motives involving coping with psychological symptoms) was author-compiled; although it was shown to have good internal consistency and convergent validity with the MMM coping with negative emotions scale in the present sample, it requires additional validation in future research. Finally, our sample included only men and women; future research is needed to investigate the current research questions among gender-diverse individuals, whom research shows are particularly vulnerable to sexual assault (Ybarra et al., 2022).

The present findings provide additional support for suggestions that sexual assault is a particularly harmful form of traumatic experience in terms of emotional consequences and extend prior research showing that sexual assault, relative to other traumatic experiences, is associated with differences in a variety of areas of cannabis use, in a pattern that is largely consistent with operant conditioning theory predictions (e.g., Stewart et al., 1998). Regardless of gender, our findings suggest that sexual assault survivors who use cannabis might benefit from exposure therapy to target their greater intrusive PTS symptoms (re-experiencing and hyperarousal), which may, in turn, motivate cannabis use to cope with these symptoms, more frequent cannabis use, and greater compulsivity craving. Indeed, prolonged exposure among substance users with PTSD has been shown to be associated with decreased substance craving over exposure sessions (Peirce et al., 2020), indicating its safety for use with trauma survivors with predominant intrusive PTS symptoms like sexual assault survivors. Moreover, our findings of several potential gender moderation effects suggest that men and women with sexual assault histories may have some differing intervention needs. While PTS Cluster D symptoms (negative alterations in mood and cognition) should be specific treatment targets in men sexual assault survivors (e.g., through interventions that target maladaptive beliefs, like cognitive restructuring), cannabis enhancement motives and purposefulness craving should be specific treatment targets in women sexual assault survivors (potentially through interventions targeting anhedonia; Craske et al., 2019).

## Data availability statement

The data analyzed in this study are subject to the following licenses/restrictions: the combined dataset generated for and analyzed in this study is available from the corresponding author upon reasonable request and pending any required ethical approvals. Requests to access these datasets should be directed to SS, sstewart@dal.ca.

## Ethics statement

The studies involving humans were approved by Nova Scotia Health Research Ethics Board. The studies were conducted in accordance with the local legislation and institutional requirements. The participants provided their written informed consent to participate in this study.

## Author contributions

SS: Writing – review & editing, Writing – original draft, Visualization, Supervision, Methodology, Funding acquisition, Formal Analysis, Conceptualization. JK: Writing – review & editing. MW: Writing – review & editing, Validation, Conceptualization. PC: Writing – review & editing, Resources, Project administration, Investigation, Data curation. SD: Writing – review & editing, Project administration, Methodology, Investigation, Funding acquisition, Data curation. PR-S: Writing – review & editing, Supervision, Methodology, Investigation, Conceptualization.

## Funding

The author(s) declare that financial support was received for the research, authorship, and/or publication of this article. This research was supported by a Cannabis and Mental Health Catalyst Grant (Principal Investigator: SS) from the Mental Health Commission of Canada (202010PJK), by two grants (Principal Investigators: SS and SD, respectively) from the Nova Scotia Health Research Fund (NSHA 1021290 and NSHA 1026566, respectively), and by funding from the Dalhousie Department of Psychiatry Research Fund (Principal Investigator: SS). JK is supported by a Doctoral Fellowship from the Social Sciences and Humanities Research Council of Canada (SSHRC). SD is supported by graduate studentships from the Chronic Pain Center of Excellence for Canadian Veterans’ Capacity Building Initiative, the L’Oreal-UNESCO & France-Canada Research Fund for Women in Science Scholarship, and the Dalhousie Medical Research Foundation’s MacQuarrie Neuroscience Research Graduate Studentship. SS is supported through a Tier 1 Canada Research Chair in Addictions and Mental Health.

## Footnotes

^1^The only difference in inclusion criteria between the two parent studies was that DeGrace et al. (2023, 2024) specified the dose per week that was required to be considered a weekly user (i.e., 1 g of cannabis used per week in the last month; see Gabrys and Porath, 2019) whereas Romero-Sanchiz et al. (2022) only required weekly use over the last month with no minimum dosage specified.

^2^We did not include sexual assault in this tally, nor did we include responses to open-ended item 17 (i.e., “other”) as it would require researcher subjectivity in determining appropriateness as a DSM-5 (American Psychiatric Association, 2013) Criterion A exposure. Each category of a traumatic event was scored as 0 or 1, and the total was tallied. Note that this score does not quantify repeated traumatic events in the same category (e.g., multiple physical assaults) as the LEC did not provide this information. We also only used personally experienced trauma in this trauma load score, as witnessing or learning about the event happening to a loved one was not included in our definition of sexual assault trauma, and we wished to maintain equivalence. It is also important to note that item 9 (other unwanted or uncomfortable sexual experiences) was included among the non-sexual assault traumas.

^3^While alpha >0.70 is typically considered the cut point for determining adequate internal consistency, since alpha is influenced by scale length, we followed recommendations that alpha >0.60 should be considered adequate in the case of short scales of six items or less (Loewenthal and Lewis, 2021).

^4^Values of 0 were not possible in our sample since participants had already been screened for regular cannabis use in the study eligibility assessment. It is also important to note that *n* = 79 rather than 100 for this variable was due to some technical problems in the administration of this item for the first approximately half of the participants in one of the two parent studies, as reported in Romero-Sanchiz et al. (2022).

^5^There were two differences between the MMM administered by Romero-Sanchiz et al. (2022) and that administered by DeGrace et al. (2023, 2024). First, the additional two coping motives items (coping with psychological symptoms) were added midway in the Romero-Sanchiz et al. (2022) study; thus, *n* = 81 rather than 100. Second, the MMM was administered on a four-point scale by DeGrace et al. (2023, 2024) and the original five-point scale by Romero-Sanchiz et al. (2022). Given the anchors were similar across studies for four of the five ratings (all save for the midpoint in Romero-Sanchiz et al. (2022) being absent in DeGrace et al. (2023, 2024)), the scores of DeGrace et al. (2023, 2024) were put onto the same scale as Romero-Sanchiz et al. (2022) by converting scores of 3–4 and 4–5 in the dataset of DeGrace et al. (2023, 2024) prior to merging of the two datasets.
